# IJCARS—IPCAI 2022 special issue: 13th international conference on information processing in computer-assisted interventions 2022—part 1

**DOI:** 10.1007/s11548-022-02648-6

**Published:** 2022-05-06

**Authors:** Shekoofeh Azizi, Alexander Seitel

**Affiliations:** 1 Google Brain, Toronto, Canada; 2 Division of Intelligent Medical Systems, German Cancer Research Center (DKFZ), Heidelberg, Germany

We are delighted to present this special issue of International Journal of Computer Assisted Radiology and Surgery (IJCARS) as part 1 of the proceedings of the 13th International Conference on Information Processing in Computer-Assisted Interventions (IPCAI) 2022. IPCAI will be held in conjunction with the Computer Assisted Radiology and Surgery (CARS) congress. The contributions contained in this special issue will be presented on June 7th and 8th during the hybrid event in Tokyo.

Since 2010, IPCAI has been a premier international and interdisciplinary forum that brings together clinicians, computer scientists, engineers and other researchers in a unique setting. From the beginning, the aim of the meeting was to foster active engagement of the attendees by providing session formats that allow for in-depth discussions of the topics presented. Putting strong focus on translational research at the interface between computational and engineering sciences and clinical and interventional practice distinguishes IPCAI from other conferences in the field. Key topics include surgical data science, interventional imaging, interventional robotics, tracking and navigation, surgical planning and simulation, augmented reality and advanced visualization as well as surgical skill analysis and workflow.

Throughout the last years, two types of contributions to IPCAI emerged: (1) *Regular Papers* as full, journal-style contributions in which authors can present their research in depth, and (2) *Long Abstracts* that are intended to showcase novel ideas, software platforms, or recent breakthroughs, including those with preliminary but limited experimental validation.

Each *Regular Paper* submission to IPCAI 2022 underwent a rigorous review process to ensure high quality among the accepted contributions which are also published as “Original Articles” in a special issue—as this one—of the IJCARS. At least three external reviewers and two area chairs judged the quality of each submission in a two-stage review process. In case of inconclusive reviews for which no straightforward decision was possible, the IPCAI program board was approached by the program chairs to decide about final acceptance to the conference. All accepted papers were then forwarded to IJCARS for a final review step typically involving minor revisions. For this year’s IPCAI, we received 46 *Regular Paper* submissions from researchers across Europe, North America and Asia. After the described review process, 29 were accepted for presentation at IPCAI and publication in IJCARS.

The *Long Abstract* submissions were reviewed by at least two reviewers based on whose judgment the program chairs formed the final decision regarding presentation at IPCAI 2022. The authors of each accepted *Long Abstract* were offered to publish their manuscript as “Short Communication” in IJCARS which then resulted as well in a final review step typically involving minor revisions. We received 12 *Long Abstract* submissions for IPCAI 2022, 10 of which were accepted for presentation at the conference.

In part 1 of the IJCARS-IPCAI 2022 special issue, we now present 16 *Regular Papers* and 1 *Long Abstract* of IPCAI 2022. A separate part 2 of this special issue will include the remaining 13 *Regular Papers* and additional *Long Abstracts*.

Scientifically, the contributions in this special issue cover a wide range of topics with machine learning being the primary common methodological denominator. Applications range from robotics, augmented reality and classical computer-assisted interventions over intraoperative imaging such as ultrasound and laparoscopy to surgical workflow and surgical data science.

Naturally, putting together such an exciting scientific program would not have been possible without the support of many dedicated individuals. We want to thank our area chairs, reviewers, and the IPCAI program board for their help throughout the whole review process and for providing valuable feedback to the authors. We acknowledge all the support we received from the IPCAI general chairs that were always open to answer our questions and provide valuable advice along the way. A special thank you goes to the CARS Office and the IJCARS Editorial Office for their support and for providing us with the opportunity to again hold IPCAI jointly with the CARS conference and publish our accepted manuscripts in IJCARS. We are furthermore deeply indebted to our sponsors that make such events possible and provide further incentives for participants by sponsoring dedicated awards. Last but not least, our deepest gratitude goes to all the authors of IPCAI 2022. Without your contributions and ongoing support, this conference is unimaginable.

Shekoofeh Azizi and Alexander Seitel

IPCAI 2022 Program Chairs

